# Does chronic pain hinder physical activity among older adults with type 2 diabetes?

**DOI:** 10.1080/21642850.2020.1807350

**Published:** 2020-08-25

**Authors:** Nanna Wackström, Anne M. Koponen, Sakari Suominen, Ina M. Tarkka, Nina Simonsen

**Affiliations:** a Folkhälsan Research Center, Public Health Research Program, Helsinki, Finland; b Health Sciences, Faculty of Sport and Health Sciences, University of Jyväskylä, Jyväskylä, Finland; c Department of Public Health, University of Helsinki, Helsinki, Finland; d Department of Public Health, University of Turku, Turku, Finland; e School of Health and Education, University of Skövde, Skövde, Sweden

**Keywords:** T2D, chronic pain, physical activity, older adults

## Abstract

**Background:** Physical activity (PA) is a key component in management of type 2 diabetes (T2D). Pain might be a barrier to PA especially among older adults with T2D, but surprisingly few studies have investigated the association between chronic pain and PA. Our aim was to evaluate the prevalence of chronic pain among older adults with T2D and to examine the association between chronic pain and PA while taking important life-contextual factors into account.

**Methods:** Data of this register-based, cross-sectional study were collected in a survey among adults with T2D (n=2866). In the current study, only respondents aged 65–75 years were included (response rate 63%, *n*=1386). Data were analysed by means of descriptive statistics and multivariate logistic regression analysis.

**Results:** In total, 64% reported chronic pain. In specific groups, e.g. women and those who were obese, the prevalence was even higher. Among respondents experiencing chronic pain, frequent pain among women and severe pain among both genders were independently associated with decreased likelihood of being physically active. Moreover, the likelihood of being physically active decreased with higher age and BMI, whereas it increased with higher autonomous motivation and feelings of energy. Among physically active respondents suffering from chronic pain, neither intensity nor frequency of pain explained engagement in exercise (as compared with incidental PA). Instead, men were more likely to exercise regularly as were those with good perceived health and higher autonomous motivation.

**Conclusions:** The prevalence of chronic pain is high among older adults with T2D. This study shows that among those suffering from chronic pain, severe pain is independently and inversely associated with being physically active, as is frequent pain, but only among women. Moreover, the findings show the importance of autonomous motivation and health variables for both incidental PA and exercise among older adults with T2D experiencing chronic pain.

## Background

Type 2 diabetes (T2D) is one of the fastest-growing chronic diseases worldwide (World Health Organization, 2016). Globally, about 425 million people had diabetes in 2017; and it is estimated that in 2045, about 629 million people, i.e. 10% of the adult population, have diabetes (International Diabetes Federation, 2017). In Finland, the prevalence of diabetes has nearly tripled in the population aged 65 years and older during the past 15 years (Kela (Social Insurance Institution of Finland), 2016), which generates public health challenges (Diabetesbarometri, 2015). Self-management is essential for effective glycemic control, and education and support in self-management are crucial in diabetes care (American Diabetes Association, 2015).

Together with medication and a healthy diet, physical activity (PA) is a key component in the management of T2D, and this extends to older adults (International Diabetes Federation, 2017, 2013). Meta-analyses, among people with T2D, have shown positive effects of regular PA on glycemic control (Boulé, Haddad, Kenny, Wells, & Sigal, 2001; Irvine & Taylor, 2009; Snowling & Hopkins, 2006; Umpierre, Ribeiro, Schaan, & Ribeiro, 2012), cardiorespiratory fitness (Boulé, Kenny, Haddad, Wells, & Sigal, 2003) and muscle strength (Irvine & Taylor, 2009). In general, people aged 65 years and older are recommended to do at least 30 min of moderate aerobic PA five times per week or at least 20 min of vigorous aerobic PA three times per week, and at least two weekly sessions each of resistance and flexibility training (Pescatello, Arena, Riebe, & Thompson, 2014). However, there is a need for individually tailored PA, especially for older adults with chronic diseases (U.S. Department of Health and Human Services, 2008; World Health Organization, 2010). For older adults with diabetes, any kind of PA, not only programmed exercise, is beneficial (Ferriolli, Pessanha, & Marchesi, 2014). Light-intensity PA has been found to be beneficially associated with several health-related factors (Buman et al., 2010; del Pozo-Cruz, Garcia-Hermoso, Alfonso-Rosa, Alvarez-Barbosa, & Owen, 2018).

However, previous studies have shown low engagement in PA among people with T2D (Hays & Clark, 1999; Morrato, Hill, Wyatt, Ghushchyan, & Sullivan, 2007; Thiel, Al Sayah, Vallance, Johnson, & Johnson, 2016; Thomas, Alder, & Leese, 2004). Qualitative studies in this patient group have identified pain as a barrier to PA (Mier, Medina, & Ory, 2007; Schoenberg & Drugle, 2001) and a relationship between chronic pain and difficulties following an exercise program has been reported (Krein, Heisler, Piette, Makki, & Kerr, 2005, 2007), though not all findings are consistent. For example, Butchart, Kerr, Heisler, Piette, and Krein (2009) found no difference in the weekly time spent engaged in PA among those with or without chronic pain.

People with diabetes are at a higher risk of pain because of disease-related factors such as peripheral neuropathy, skin tears, depression or falls (International Diabetes Federation, 2013). Also, musculoskeletal pain seems to be more common among people with T2D than in the general population and associated with physical inactivity, lower physical functioning and quality of life, and higher BMI (Molsted, Tribler, & Snorgaard, 2012). Overall, chronic pain negatively affects daily activities and quality of life, causing complications like anxiety, depression, sleep disturbances and disability (Dueñas, Ojeda, Salazar, Mico, & Failde, 2016; Geneen et al., 2017). Earlier studies also largely suggest that there is a strong negative association between chronic pain and PA (Dueñas et al., 2016). As the prevalence of chronic pain increases with age (Fayaz, Croft, Langford, Donaldson, & Jones, 2016; Landmark, Romundstad, Borchgrevink, Kaasa, & Dale, 2011), chronic pain might be a barrier to PA especially among older adults with T2D. However, few studies have explored the prevalence of chronic pain in this age group of T2D patients. Moreover, research on the association between chronic pain and PA among older adults with T2D is scarce.

Pain is defined as ‘an unpleasant sensory and emotional experience associated with actual or potential tissue damage, or described in terms of such damage’ (International Association for the Study of Pain, 1986). Pain is always subjective and something one relates to through earlier experiences (International Association for the Study of Pain, 1986), wherefore self-report is the most reliable method to assess pain (Connelly, 1998; Ho, Spence, & Murphy, 1996). Pain can be determined by unidimensional scales, where different aspects of pain – for example, pain frequency and intensity – are judged separately (Ho et al., 1996).

Chronic pain has been described as pain that lasts beyond the normal healing process (Treede et al., 2015, referring to Bonica 1953). Usually, pain is classified as chronic when it has lasted for over three or six months (Merskey & Bogduk, 1994). Nowadays the biopsychosocial model is the most broadly accepted approach to chronic pain. Chronic pain is seen as a multidimensional phenomenon, influenced by an interaction between physiological, psychological and social factors (Gatchel, Peng, Peters, Fuchs, & Turk, 2007).

In addition to pain, other personal and environmental factors influence engagement in PA. The PA level tends to decrease with age, men tend to be more physically active than women, and people with higher education tend to be more physically active than people with lower education (Choi, Lee, Lee, Kang, & Choi, 2017). Poor health or fitness (Choi et al., 2017), overweight (Trost, Owen, Bauman, Sallis, & Brown, 2002) and lack of energy (Eyler et al., 2002) may hinder PA, whereas social support tends to be positively associated with PA (Choi et al., 2017). Autonomous motivation for PA, defined by the self-determination theory (SDT), has been shown to be strongly positively related to engagement in PA (Koponen, Simonsen, & Suominen, 2017; Teixeira, Carraça, Markland, Silva, & Ryan, 2012) and success in increasing PA (Koponen, Simonsen, & Suominen, 2018). In line with the biopsychosocial model (Gatchel et al., 2007), social support, perceived energy, good perceived health and autonomous motivation may also be seen as resilience factors modulating the pain experience. Earlier studies among older adults with chronic diseases have, for example, found that higher levels of self-efficacy reduced the association between chronic pain and difficulty exercising (Krein, Heisler, Piette, Butchart, & Kerr, 2007). Moreover, negative mood and fear of pain, experienced by many patients with chronic pain, may reduce treatment motivation and lead to inactivity (Gatchel et al., 2007). However, autonomously motivated people might be less likely to give up PA when facing barriers such as pain.

A broad understanding of factors that influence engagement in PA is important when supervising and motivating older adults with T2D in PA. Chronic pain might be one barrier; however, little is known about the relationship between chronic pain and PA among people with diabetes. Moreover, a better understanding of how various factors, such as socio-demographic and psychosocial variables – in line with the biopsychosocial view on pain – may affect this relationship is needed. Our aim was to evaluate the prevalence of chronic pain among older adults with T2D living in Finland and to examine the association between chronic pain, its frequency and intensity, and engagement in PA. First, we examined a possible relationship between the presence of chronic pain and engagement in PA. Second, we examined whether there is a relationship between (a) pain frequency and engagement in PA, and (b) pain intensity and engagement in PA among those reporting chronic pain. Third, we examined whether there is a relationship between (a) pain frequency and engagement in exercise, and (b) pain intensity and engagement in exercise among those who had chronic pain and were physically active. We took into account several potentially confounding socio-demographic and life-contextual factors (gender, age, educational level, body mass index (BMI), perceived health, autonomous motivation, feelings of energy and social support in diabetes care) that have been found to affect engagement in PA directly, but which also may affect engagement in PA indirectly via pain experience.

## Methods

### Study design

This study is part of a larger study of quality of care in T2D. Cross-sectional data were collected via a postal survey. The sample was accrued from the register of the Social Insurance Institution of Finland (Kela). Kela is a Finnish government agency in charge of settling benefits under national social security programs and is, via legislation, authorized to hold a nationwide register of people having an entitlement to special reimbursement for medicine expenses because of chronic disease.

The inclusion criteria for participation in this study were:

1. Entitlement to a special reimbursement for medicines used in the treatment of T2D (ICD-10 code E11) in 2000–2010, and the right was valid in September 2011 and onward,
2. Born between 1936–1991 (20–75 years) and alive,
3. Finnish as native language,
4. Living in one of the five study municipalities.

The inclusion criteria were fulfilled by 7575 persons. Based on power-analysis, a sample of 5167 persons was collected by Kela: 2000 persons from the two large municipalities and all persons from the three small municipalities. The sample consisted of 2962 (57%) men and 2205 (43%) women, which corresponded to the gender rates in the total population of persons with T2D in the study municipalities. The questionnaires were sent per postal mail by Kela. After two reminders the final response rate was 56% (*n* = 2866). Respondents aged 65–75 years were included in the present analysis (*n* = 1386).

### Ethics statement

The research plan was accepted by the Ethical Committee of the Hjelt Institute, University of Helsinki, and permission to conduct the study was received from Kela. The questionnaires were provided only by an identification number, which was needed in order to check for non-response. The identity of respondents was not revealed to the researchers at any point of the sample or data collection. The respondents gave their consent to participate by the act of returning the questionnaire.

### Respondents

The response rate in the age group 65–75 years was 63%. The mean age among the respondents was 69.4 years (SD = 3.3 years) and the mean duration of diabetes was 9.4 years (SD = 6.8 years). Of the respondents, 54.5% were men. Municipal primary care health center was the primary care place in diabetes care for 93.5% of the respondents. The majority (77.8%) used oral diabetes medication only. Almost all (96.8%) had at least one other chronic disease or condition apart from diabetes, and nearly half (47.0%) had at least one diabetes-related complication. Hypertension (57.5%) was the most common chronic disease or condition, and retinopathy (22.7%) was the most common diabetes-related complication. Of the respondents, 9.9% reported having neuropathy. Moreover, 9% reported that they could not be physically active due to a disease or disability. Still, 25% of these respondents were physically active according to the definition used in this study.

### Study variables

#### Dependent variables

*Engagement in PA* was assessed by the question ‘How physically active are you during leisure time? If the activity differs much between seasons, choose the alternative that best describes your average activity level’ (Helakorpi, Patja, Prättälä, Aro, & Uutela, 2002). Response options were ‘during leisure time I usually sit and perform tasks, where I do not move or that are not physically demanding’, ‘during leisure time I either walk, bicycle or do other activities (such as gardening) at least four hours per week’, ‘during leisure time I perform health exercise at least three times per week’ and ‘during leisure time I train regularly several times per week in order to participate in competitions’. The question assesses PA in general, without indication of a time period. Respondents who chose the first response option were classified as physically inactive. All the others were classified as physically active.

*Engagement in exercise* was assessed by the same question as engagement in PA. Respondents who chose the response alternative ‘during leisure time I either walk, bicycle or do other activities (such as gardening) at least four hours per week’ were classified as engaging in incidental PA. Respondents who chose either the response option ‘during leisure time I perform health exercise at least three times per week’ or ‘during leisure time I train regularly several times per week in order to participate in competitions’ were classified as engaging in exercise. In further analyses among all the physically active respondents, those who engaged in incidental PA were compared with those who engaged in exercise.

#### Main independent variables

The main independent variables consisted of three variables assessing pain. The questions were asked of all respondents, and all questions had a response option suitable for those not experiencing pain. *Presence of chronic pain* was determined based on the question ‘If you have pain, when did it start?’. The question was based on the International Association for the Study of Pain’s (IASP) (International Association for the Study of Pain, 1986) classification of chronic pain. Response options were ‘under 1 month ago’, ‘1–3 months ago’, ‘over 3 months to 6 months ago’, ‘over 6 months ago’ and ‘does not apply to me’. Respondents reporting that their pain started ‘over 3 months to 6 months ago’ or ‘over 6 months ago’ were classified as having chronic pain.

*Pain intensity* was assessed by the question ‘How severe pain have you had during the past four weeks?’. The question was based on the Finnish validated version of the Rand-36-item survey (Aalto, Aro, & Teperi, 1999). Response options were ‘none’, ‘very mild’, ‘mild’, ‘moderate’, ‘severe’ and ‘very severe’. The variable was categorized into: (1) mild (mild, very mild or no pain), (2) moderate, and (3) severe (severe or very severe). This categorization has been found useful for identifying persons with pain of a more complex nature (Jensen, Sjøgren, Ekholm, Rasmussen, & Eriksen, 2004).

We also included a question about pain frequency as pain intensity and pain frequency are suggested to represent distinct dimensions of pain (Denkinger, Lukas, Nikolaus, Peter, & Franke, 2014). *Pain frequency* was assessed by the question ‘Do you have pain?’. Response options were ‘no’, ‘seldom’, ‘sometimes’, ‘often’, ‘daily’ and ‘all the time’. This variable was dichotomized into (1) often (often, daily or all the time), and (2) seldom (seldom or sometimes).

#### Additional independent variables measuring health and wellbeing

We included physical health variables (BMI and perceived health), psychological variables (autonomous motivation and energy) and a social support variable (social support in diabetes care) in the analyses.

*BMI* was calculated by dividing weight in kilograms by the square of height in meters. Weight was assessed by the question ‘Approximately how much do you weigh with light clothes?’ and height was assessed by the question ‘Approximately how tall are you?’.

*Perceived health* was measured by a single-item scale (1 = excellent, 2 = very good, 3 = good, 4 = quite poor, 5 = poor). This variable was dichotomized into: (1) poor perceived health (4–5), and (2) good perceived health (1–3). Perceived health measured with a single-item question has been found to be a valid indicator of overall health (Schnittker & Bacak, 2014).

For autonomous motivation, energy and social support average sum scales were calculated. *Autonomous motivation* was measured by The Autonomous Regulation Scale B, which consists of five items from the validated Treatment Self-Regulation Questionnaire (range: 1 = not at all true, 7 = very true) (Self-determination theory). The scale assesses respondents’ autonomous motivation to eat healthy in general and to exercise, without indication of a time period. Example item: ‘I follow a healthy diet and exercise regularly because I personally believe that these are important for staying healthy’. Cronbach’s α was 0.83.

*Energy* was measured by a 4-item scale from the validated Rand-36-item survey. The scale measures energy during the past four weeks (range: 0–100%) (Aalto et al., 1999; Hays, Sherbourne, & Mazel, 1993). Cronbach’s α was 0.85.

*Social support in diabetes care* was measured using a 12-item scale that measures support and help received from health care personnel, relatives and friends (range: 1 = fully disagree, 5 = fully agree) in general (Toljamo, 1999). The scale is based on social support scales by Brandt and Weinert (1981), Goodenow, Reisine, and Grady (1990), Norbeck, Lindsay, and Carrieri (1981; 1983), Stewart and Tilden (1995) and Weinert (1987). Examples of items are ‘My family and friends take care of things for me when needed’, and ‘I get the information I need from the health care personnel’. Cronbach’s α was 0.75.

Moreover, we included the following demographic variables: gender, age and educational level. *Educational level* was assessed by the question ‘What is your professional education?’. This variable was dichotomized into (1) lower education (vocational, upper secondary education or less) and (2) higher education (college, polytechnic or university).

#### Descriptive background variables

Several background variables were utilized in descriptive analyses to provide basic information about the study sample. Variables such as diabetes duration, diabetes medication, diabetes-related complications, other chronic diseases or conditions, and use of pain medication were chosen. PA guidance was assessed by two questions: ‘Have you received recommendations to perform regular PA?’ (‘yes’ or ‘no’) and ‘In your primary care place, have you received information and guidance about PA suitable for you?’ (‘no’, ‘too little’, ‘enough’ or ‘does not apply to me’). Pain location was assessed by the question ‘If you have pain, where does it primarily occur?’. For this question, seven response options were given (‘head or facial area’, ‘neck or shoulders’, ‘one or both arms’, ‘one or both legs’, ‘abdominal area’, ‘low back’, ‘does not apply to me’) and one open-response option (‘somewhere else’) (Saastamoinen, Leino-Arjas, Laaksonen, Martikainen, & Lahelma, 2006).

#### Statistical analysis

In descriptive analyses, baseline associations between independent, background and dependent variables were tested with cross-tabulation and chi-square (*χ*^2^) test. Moreover, correlations between study variables were explored by Pearson’s or Spearman’s correlation in order to avoid multicollinearity problems in the multivariate logistic regression models. The relationship between chronic pain and engagement in PA was tested in bivariate logistic regression analyses, and adjusted for demographic, physical, psychological and social support variables in multivariate logistic regression analyses. Odds ratio (OR) with 95% confidence interval (CI) are reported. The first set of analyses included the whole sample (in order to compare those with chronic pain and those without chronic pain, *n* = 1177, missing 15.1%) and the relationship between the presence of chronic pain and engagement in PA was tested. In the second set of analyses, only respondents with chronic pain were included (*n* = 757) as we were specifically interested in the association of the frequency and intensity of chronic pain with PA. In these analyses, the relationship between (a) pain frequency and engagement in PA, and (b) pain intensity and engagement in PA was tested in different models because there was a quite strong correlation between pain frequency and pain intensity (0.53, *p* < 0.001). In the third set of analyses, only respondents who had chronic pain and were physically active were included (*n* = 530) and the relationship between (a) pain frequency and engagement in exercise, and (b) pain intensity and engagement in exercise was tested.

In all three sets of analyses, the relationship between the main independent variable and PA was first tested separately in bivariate logistic analyses. Thereafter, all the other independent variables were added to the multivariate model at the same time. Earlier studies have shown gender differences in the association between pain and PA [e.g. Landmark et al., 2011]. Thus, we tested the interaction term between gender and pain in all fully adjusted models of PA. The interaction term between gender and pain frequency on engagement in PA was the only one that was statistically significant (*p* < 0.05).

The independent variables measuring health and wellbeing were chosen based on previous research. These variables may affect engagement in PA directly, but also indirectly through pain experience. From a larger number of possible variables, the final ones were chosen based on correlations. Correlations were calculated in the whole sample, and among respondents with chronic pain separately. Of physical health variables (BMI, diabetes complication, other chronic disease or condition and perceived health) and psychological variables (autonomous motivation, depression, energy and psychological well-being), the two variables that correlated strongest with PA were chosen for final analyses. Moreover, all demographic variables and the social support variable were included in final analyses. Thus, besides the main independent variables, i.e. the variables assessing pain, additional independent variables chosen for final analyses were gender, age, education level, BMI, perceived health, autonomous motivation, energy and social support in diabetes care. All statistical analyses were performed using SPSS version 24. Listwise deletion was used for missing values. The level of statistical significance was set at *p* < .05.

## Results

Chronic pain, i.e. pain that had lasted for over three months, was reported by 64.3% of the respondents. Among almost all of these respondents (94.3%), the pain had lasted for over six months. Chronic pain was more common among women than men, and among those who had at least one diabetes-related complication or other chronic disease or condition compared to those who did not have these. The prevalence of chronic pain increased with higher numbers of chronic diseases or conditions and higher BMI. Chronic pain was more common among those reporting poor perceived health compared to those reporting good perceived health. A larger number of those with neuropathy had chronic pain than those without neuropathy (Table 1).

**Table 1.** Prevalence of chronic pain by different background variables (*N* = 1177^a^).

	Chronic pain, % (*n*)	*p*-value
Whole sample	64.3 (757)	
Gender Man Woman	58.3 (376) 71.6 (379)	<0.001***
	
Age (years) 65–69 70–75	65.3 (419) 63.2 (338)	0.457	
Diabetes duration (years) < 5 5–10 > 10	62.4 (166) 64.4 (299) 62.6 (239)	0.803	
Education level Lower Higher	64.3 (454) 63.6 (281)	0.802	
BMI (kg/m^2^) < 25 25.0–29.9 ≥ 30	58.0 (109) 60.7 (281) 70.1 (340)	0.001**	
Diabetes medication Only tablets Other	64.0 (563) 63.7 (165)	0.936	
Diabetes complication Yes No	69.4 (365) 59.2 (351)	<0.001***	
Other chronic disease or condition Yes No	65.1 (736) 37.1 (13)	0.001**	
Amount of other chronic diseases or conditions 0 1 2 3 ≥ 4	30.8 (8) 40.5 (30) 45.8 (65) 61.8 (107) 74.6 (259)	<0.001***	
Perceived health Poor Good	73.6 (452) 53.7 (297)	<0.001***	
Neuropathy Yes No	84.0 (105) 62.0 (652)	<0.001***	

Notes: Differences between groups are tested by *χ*^2^ test. Results are given as the proportion [% (*n*)] of respondents that had chronic pain in the different groups.

**p < 0.01, ***p < 0.001.

^a^Respondents in the whole study sample who answered the question about presence of chronic pain.

Of respondents with chronic pain, more than half (54.5%) reported that they had pain often, and 16.6% reported that their pain had been severe during the past four weeks (Table 2). Pain medication was used daily by 21.0% and when necessary by 60.2%. The most common pain locations were legs (55.7%), low back (39.9%), and neck and shoulders (33.8%).

**Table 2.** Distribution of pain frequency (*N* = 701) and pain intensity (*N* = 706) among respondents with chronic pain.

		Respondents with chronic pain		
		All, % (*n*)	Physically active, % (*n*)	Physically inactive, % (*n*)
*Pain frequency*				
Seldom		45.5 (319)	49.9 (259)	33.0 (60)
Often		54.5 (382)	50.1 (260)	67.0 (122)
Total		100.0 (701)	100.0 (519)	100.0 (182)
*χ*^2^			15.587, *p* < 0.001	
*Pain intensity*				
Mild		36.8 (260)	40.0 (209)	27.7 (51)
Moderate		46.6 (329)	46.8 (244)	46.2 (85)
Severe		16.6 (117)	13.2 (69)	26.1 (48)
Total		100.0 (706)	100.0 (522)	100.0 (184)
*χ*^2^			19.211, *p* < 0.001	

Note: Seldom: pain seldom or sometimes; Often: pain often, daily or all the time, Mild: mild or very mild pain during the past four weeks; Severe: severe or very severe pain during the past four weeks.

The majority (88.5%) had been advised to be physically active regularly, but less than half (48.7%) thought that they had received enough information about PA suitable for them. A lower percentage of those who had chronic pain (74.0%) were physically active compared to those who did not have chronic pain (81.6%) (*p* = 0.004). Of the respondents who had chronic pain and were physically active, 73.6% engaged in incidental PA, 25.8% in exercise, and 0.6% trained in order to participate in competitions. Compared to the physically inactive, a lower percentage of those who were physically active reported that they had pain often (*p* < 0.001) and that their pain had been severe during the past four weeks (*p* < 0.001) (Table 2).

In the whole sample there was a negative, but weak, correlation between presence of chronic pain and PA (−0.09, *p* < 0.01). Presence of chronic pain correlated most strongly with energy (−0.23, *p* < 0.001) and perceived health (−0.21, *p* < 0.001). Among respondents with chronic pain, pain frequency and pain intensity correlated most strongly with each other (0.53, *p* < 0.001), with energy (−0.27, *p* < 0.001 and −0.28, *p* < 0.001, respectively) and perceived health (−0.24, *p* < 0.001 and −0.21, *p* < 0.001, respectively). The correlation between pain frequency and PA was −0.15 (*p* < 0.001), as was the correlation between pain intensity and PA. Regarding PA, both in the whole sample and among those with chronic pain, the strongest correlations were seen with autonomous motivation (0.31, *p* < 0.001 and 0.31, *p* < 0.001), BMI (−0.29, *p* < 0.001 and −0.31, *p*<0.001) and energy (0.24, *p* < 0.001 and 0.22, *p* < 0.001).

### Primary analyses

In the whole sample, the bivariate logistic regression analysis showed a significant negative relationship between presence of chronic pain and engagement in PA (*p* = 0.004). After adjusting for the other independent variables, however, chronic pain was not related to engagement in PA. The odds of being physically active decreased with higher age and BMI, and increased with higher autonomous motivation and feelings of energy (Table 3).

**Table 3.** Relationship between presence of chronic pain and engagement in PA in the whole study sample, and between frequency and intensity of pain and engagement in PA among respondents with chronic pain.

				Women		Men				
	OR (95% CI)	*P*		OR (95% CI)	*p*	OR (95% CI)	*p*		OR (95% CI)	*p*
*Bivariate model* (*N = 1133^a^*)				*Bivariate model* (*N = 353^b^*)		*Bivariate model* (*N = 346*^b^)		*Bivariate model* (*N = 706^b^*)		
**Chronic pain** No Yes	1.000 0.642 (0.474–0.870)	0.004**	**Pain frequency** - Seldom - Often	1.000 0.309 (0.173–0.553)	<0.001***	1.000 0.680 (0.420–1.099)	0.115	**Pain intensity** - Mild - Moderate - Severe	1.000 0.700 (0.473–1.038) 0.351 (0.217–0.566)	0.076 <0.001***
*Multivariate model* *(N = 997^a^)*				*Multivariate model* *(N = 315^b^)*		*Multivariate model* *(N = 308^b^)*		*Multivariate model* *(N = 625^b^)*		
**Chronic pain** No Yes	1.000 0.851 (0.587–1.234)	0.395	**Pain frequency** - Seldom - Often	1.000 0.335 (0.160–0.701)	0.004**	1.000 0.972 (0.540–1.747)	0.924	**Pain intensity** - Mild - Moderate - Severe	1.000 0.870 (0.533–1.420) 0.507 (0.276–0.931)	0.577 0.029*
Gender Woman Man	1.000 0.948 (0.666–1.349)	0.767						Gender - Woman - Man	1.000 0.794 (0.515–1.225)	0.298
Age	0.890 (0.844–0.939)	<0.001***	Age	0.894 (0.812–0.986)	0.024*	0.927 (0.845–1.018)	0.112	Age	0.910 (0.853–0.972)	0.005**
Education level Lower Higher	1.000 1.387 (0.969–1.986)	0.074	Education level - Lower - Higher	1.000 1.137 (0.593–2.182)	0.699	1.000 1.488 (0.812–2.728)	0.198	Education level - Lower - Higher	1.000 1.456 (0.936–2.267)	0.096
BMI	0.872 (0.843–0.902)	<0.001***	BMI	0.848 (0.797–0.902)	<0.001***	0.872 (0.822–0.926)	<0.001***	BMI	0.862 (0.826–0.899)	<0.001***
Perceived health Poor Good	1.000 1.112 (0.750–1.650)	0.597	Perceived health - Poor - Good	1.000 1.021 (0.490–2.127)	0.957	1.000 1.464 (0.739–2.900)	0.275	Perceived health - Poor - Good	1.000 1.307 (0.797–2.144)	0.288
Autonomous motivation	1.644 (1.422–1.901)	<0.001***	Autonomous motivation	1.798 (1.366–2.367)	<0.001***	1.437 (1.136–1.817)	0.003**	Autonomous motivation	1.600 (1.332–1.921)	<0.001***
Energy	1.017 (1.007–1.026)	<0.001***	Energy	1.020 (1.004–1.036)	0.015*	1.015 (0.999–1.032)	0.065	Energy	1.016 (1.005–1.028)	0.004**
Social support	0.887 (0.650–1.209)	0.448	Social support	0.806 (0.517–1.255)	0.339	0.690 (0.435–1.095)	0.116	Social support	0.676 (0.460–0.991)	0.045*
Nagelkerke *R*^2^ = 0.296				Nagelkerke *R*^2^ = 0.385		Nagelkerke *R*^2^ = 0.254		Nagelkerke *R*^2^ = 0.311		

Notes: The relationship between the frequency of pain and engagement in PA is presented for women and men separately. Bivariate and multivariate logistic regression analysis.

OR: odds ratio; CI: confidence interval; OR in reference group = 1.000.

*p<0.05, **p < 0.01, ***p < 0.001.

^a^Respondents in the whole study sample who answered both the question about presence of chronic pain and the question about engagement in PA.

^b^Respondents with chronic pain.

In analyses among respondents suffering from chronic pain, a significant relationship between higher pain frequency and a lower likelihood of being physically active was seen in both the bivariate and the multivariate logistic regression analysis. The interaction term between gender and pain frequency was statistically significant (*p* = 0.026) and thus the findings are shown separately for women and men. The frequency of pain was associated with PA only among women. According to the multivariate model, the odds of being physically active were three times (1/0.335 = 2.985) lower among women who had pain often compared to those who had pain seldom (*p* = 0.004). Moreover, among women, the likelihood of being physically active decreased with higher age and BMI, but increased with higher autonomous motivation and feelings of energy (Table 3).

Moreover, among those with chronic pain, a significant relationship between higher pain intensity and a lower likelihood of being physically active was seen in both the bivariate and the multivariate logistic regression analysis. According to the multivariate model, the odds of being physically active were nearly two times (1/0.507 = 1.972) lower among those who reported severe pain compared to those who reported mild pain (*p* = 0.029). No difference in the likelihood of being physically active was seen between those who reported moderate pain and those who reported mild pain. In this model, as well, the odds of being physically active increased with higher autonomous motivation and feelings of energy and decreased with higher age and BMI. In addition, it decreased with higher level of social support (Table 3).

Among respondents who had chronic pain and were physically active, there was no significant association between either pain frequency or pain intensity and engagement in exercise (as compared with engaging in incidental PA). Engagement in exercise was more likely among men than among women, and more likely among respondents rating their health as good than among those rating their health as poor. The likelihood of engaging in exercise also increased with higher autonomous motivation but decreased with higher levels of social support (Table 4).

**Table 4.** Relationship between frequency and intensity of pain and engagement in exercise among respondents who had chronic pain and were physically active.

	OR (95% CI)	*p*		OR (95% CI)	*p*
*Bivariate model* *(N = 519)*			*Bivariate model* *(N = 522)*		
**Pain frequency** Seldom Often	1.000 0.848 (0.573–1.255)	0.410	**Pain intensity** Mild Moderate Severe	1.000 0.954 (0.626–1.454) 1.064 (0.577–1.961)	0.826 0.842
*Multivariate model* *(N = 464)*			*Multivariate model* *(N = 465)*		
**Pain frequency** Seldom Often	1.000 1.075 (0.677–1.707)	0.760	**Pain intensity** Mild Moderate Severe	1.000 1.103 (0.679–1.792) 1.196 (0.588–2.432)	0.693 0.621
Gender Woman Man	1.000 1.576 (1.002–2.477)	0.049*	Gender Woman Man	1.000 1.611 (1.027–2.527)	0.038*
Age	0.963 (0.897–1.033)	0.288	Age	0.975 (0.910–1.045)	0.480
Education level Lower Higher	1.000 1.231 (0.785–1.931)	0.366	Education level Lower Higher	1.000 1.228 (0.785–1.921)	0.369
BMI	1.038 (0.986–1.093)	0.152	BMI	1.043 (0.991–1.098)	0.107
Perceived health Poor Good	1.000 1.978 (1.201–3.257)	0.007**	Perceived health Poor Good	1.000 1.835 (1.120–3.006)	0.016*
Autonomous motivation	1.597 (1.261–2.021)	<0.001***	Autonomous motivation	1.567 (1.240–1.981)	<0.001***
Energy	1.011 (0.997–1.025)	0.117	Energy	1.012 (0.998–1.026)	0.087
Social support	0.549 (0.359–0.840)	0.006**	Social support	0.577 (0.378–0.881)	0.011*
Nagelkerke *R*^2^ = 0.119			Nagelkerke *R*^2^ = 0.113		

Notes: Bivariate and multivariate logistic regression analysis. OR: odds ratio; CI: confidence interval; OR in reference group = 1.000.

*p< 0.05, **p < 0.01, ***p < 0.001

## Discussion

We evaluated the prevalence of chronic pain among older adults with T2D, and examined the relationship between chronic pain and engagement in PA. The prevalence of chronic pain was high, reported by 64% of the respondents. In specific groups, such as among women, those who were obese, had neuropathy, rated their health as poor, or had four or more chronic diseases or conditions apart from diabetes, the prevalence was even higher. The prevalence of chronic pain in our sample of older adults with T2D was thus in line with numbers reported by Krein et al. (2005; 2007) and Butchart et al. (2009), but somewhat higher than numbers reported by Sudore et al. (2012). All of these studies, however, were not limited to older adults, and the pain was classified as chronic when it had lasted for six months. We classified the pain as chronic when it had lasted for over three months. However, the pain had lasted for over six months among almost all (94%) of those who had chronic pain.

To our knowledge, this is one of the first studies exploring the association between the frequency and intensity of chronic pain and being physically active among older adults with T2D. By and large, we found that those who experienced chronic pain were more often physically inactive as compared with those without chronic pain. Still, 74% of the respondents with chronic pain engaged in at least incidental PA. Among those without chronic pain, the corresponding number was 82%. These rates of engaging in PA are generally higher than in previous studies among people with T2D. In previous studies (Hays & Clark, 1999; Morrato et al., 2007; Thiel et al., 2016; Thomas et al., 2004), varied measures were used to assess PA among people with diabetes. For example, reaching the recommended amount of moderate to vigorous PA was examined (Morrato et al., 2007; Thiel et al., 2016). For older adults with diabetes, also other types of PA than planned exercise is beneficial (Ferriolli et al., 2014), wherefore we decided to classify also those respondents who engaged in incidental PA – determined as the participant either walking, bicycling or doing other activities (such as gardening) at least four hours a week – as physically active. This could explain the higher engagement in PA in our study compared to previous studies. Of the physically active respondents with chronic pain, a minority (26%) engaged regularly in exercise. Among people with diabetes in general, as well, those who are physically active tend to engage in low-intensity PA (Thomas et al., 2004). In the study by Thomas et al. (2004), 34% of the respondents had engaged in PA during the past two weeks, but among the majority (51%) of these, no change in heart rate or breathing occurred.

More specifically, our findings showed that frequent chronic pain, among women, as well as severe chronic pain, among both women and men, was related to a decreased likelihood of being physically active. The International Diabetes Federation (IDF) (2013) suggests that pain assessment should become a routine in diabetes care. Pain assessment is crucial for optimal pain management and may be particularly important among older adults with diabetes, since pain may be both underreported and undertreated in this group of patients (International Diabetes Federation, 2013). Our findings suggest further that comprehensive assessments of pain should be used when giving PA guidance. It would be especially important to identify individuals who experience frequent and severe chronic pain. In general, PA is recommended for patients with chronic pain, and it may reduce pain severity and improve physical functioning (Geneen et al., 2017). Still, many patients with diabetes choose inactivity and sedentary activities as pain management strategies (Butchart et al., 2009). This may partly be due to anxiety and fear that PA might increase their pain (Gatchel et al., 2007). Assessments should thus include feelings related to the pain experience.

Moreover, though the majority (89%) of respondents with chronic pain had been recommended to be physically active regularly, less than half (49%) considered having received enough information about personally suitable PA. This highlights the importance of both individualized PA guidance and better assessment of pain – including factors affecting the pain experience – in this patient group. Older adults with diabetes are a heterogeneous patient group (American Diabetes Association, 2015). Chronic pain, other chronic diseases or diabetes-related complications, such as diabetic foot ulcers and poor circulation in lower extremities, may limit engagement in PA, especially the performance of weight-bearing activities. Addressing chronic pain besides disease-specific issues is necessary to improve patient well-being and quality of life (Butchart et al., 2009).

Interestingly, our study showed that among older adults with chronic pain, neither pain frequency nor pain intensity explained the probability of engaging in exercise as compared with incidental PA. Instead, in addition to autonomous motivation, perceived health seems to play a role, i.e. those with good perceived health were more probable to engage in exercise, as were men. Earlier studies among patients with T2D show that women tend to prefer lower-intensity PA than men, and it has been suggested that the reasons for the gender difference might include possible physiological differences as well as less accessibility to preferred types of exercising (Miller, Gilligan, Herlache, & Regensteiner, 2012).

Overall, the current study suggests that the negative association between frequency and intensity of chronic pain and engagement in PA among older adults with T2D might be reduced by factors such as autonomous motivation and feelings of energy or vitality. This finding can be linked to the biopsychosocial perspective on chronic pain (Gatchel et al., 2007), according to which it is possible to affect the pain experience through psychosocial factors. Chronic pain did not eliminate the strong relationship between autonomous motivation and PA. Thus, in line with earlier studies (Koponen, Simonsen & Suominen, 2017, 2018; Teixeira et al., 2012), our findings suggest that supporting autonomous motivation for PA – by fostering feelings of competence, autonomy and relatedness (Ryan & Deci, 2000) – could be beneficial also among older adults with T2D suffering from chronic pain. When patients have internalized the importance of PA for health, they may be physically active, despite even experiencing frequent or more severe pain.

Moreover, feelings of energy were positively related to PA and negatively related to chronic pain. Lack of energy could, via a biopsychosocial perspective (Gatchel et al., 2007), be seen as a psychological factor related to chronic pain. According to the gate control theory (Melzack & Wall, 1965) and the neuromatrix theory of pain (Melzack, 1999, 2001) – theoretical frameworks for the biopsychosocial perspective of chronic pain – energy could be an emotional factor that influences and modulates the pain experience, for example by increasing the pain inhibitory effect. In our study, energy was also strongly related to perceived health. This is in line with a previous study (Molarius & Janson, 2002), in which tiredness/weakness was strongly related to poor self-rated health at the population level. Whether interventions promoting perceived health, i.e. focusing on both physical, mental and social health, also increase energy levels among older adults with T2D would be worth studying.

Social support from e.g. family and friends has in previous studies been positively related to engagement in PA, also in samples limited to older adults (Choi et al., 2017). Our study showed a positive correlation between social support in diabetes care and PA. However, in the multivariate regression models on pain intensity and PA, and especially regarding engagement in exercise, the relationship was significantly negative. A reason for this may be that social support also correlated positively with autonomous motivation and energy, which in turn were variables that correlated more strongly with PA. The positive correlation between social support and PA in bivariate analysis might thus be a result of the effect of other factors, such as autonomous motivation and energy. However, the correlations between the variables were moderate, which does not indicate multicollinearity problems in the multivariate regression models. Individuals who need more social support may have limited ability to perform diabetes self-care, including PA and probably especially exercise, and thus the likelihood of being physically active may be lower among them compared to those who need less social support. Another type of social interaction – social control – may also have an impact on PA together with social support. Studies have, for example, found negative associations between social control and engagement in PA among older adults (Cotter, 2012; Khan, Stephens, Franks, Rook, & Salem, 2012; Newsom, Shaw, August, & Strath, 2018), and inconsistent results regarding the interactive effect of social support and social control on PA (Fekete, Stephens, Druley, & Greene, 2006; Khan et al., 2012). High levels of both social support and social control might promote engagement in PA, but can also give conflicting messages, which may result in less PA (Fekete et al., 2006; Khan et al., 2012).

### Strengths and limitations

Due to the cross-sectional design, the direction of the observed associations cannot be confirmed and associations may also be two-sided. A high BMI, for example, can be a consequence of lack of PA, but also a barrier to engagement in PA. Chronic pain has been identified as a barrier to PA (Mier et al., 2007; Schoenberg & Drugle, 2001), but at the same time, PA might relieve chronic pain (Geneen et al., 2017). In addition, a one-time assessment of pain might not capture fluctuations of pain intensity very well. Including assessments of recalled worst pain intensity could give additional important information on the pain experience (Jensen et al., 2015). Still, a single rating of recalled average pain intensity with a verbal rating scale is considered a valid instrument for identifying persons suffering from pain, also of a more complex nature (Jensen et al., 2004).

Strengths of this study were the representative sample and a high response rate. With self-report measures it is possible to study large sample sizes at low cost, but there are also limitations related to these measures (Dale, Welk, & Matthews, 2002). Regarding self-report of BMI, there might be underestimations, and regarding PA, over-reporting of PA, especially of the amount of vigorous PA has been identified as a common phenomenon (Sallis & Saelens, 2000). On the other hand, physically inactive people tend to classify themselves as inactive (Matthews, 2002) and with self-report questionnaires it is possible to determine general categories of PA, such as low, moderate and high (Haskell, 2012). In our study, PA was assessed by one question, which assesses PA on a general level. The question has been used previously in Finnish studies, and it estimates quite well PA on the population level (Fogelholm, 2016). However, it does not take into account the intensity of PA performed. Therefore, it is difficult to directly compare our results with results from studies in which the achievement of PA recommendations has been examined. Based on the question it is possible to separate those who do not perform PA from those who perform at least some type of PA. Physical inactivity is a global health risk (World Health Organization, 2010) and it is therefore especially important to identify physically inactive individuals.

Another strength of our study was that we were able to adjust for a wide variety of important potentially confounding socio-demographic and life-contextual factors. The variables that were included in the multivariate logistic regression analyses explained approximately 30% of the likelihood of being physically active. The aim of this study was to examine the relationship between chronic pain and PA among older adults with T2D, and further to observe whether variables, which were previously found important for engagement in PA, affect this relationship. However, we were also interested in how experiencing chronic pain might affect previously found relationships between important variables, such as autonomous motivation, and engagement in PA. More research, including long-term intervention studies, is needed for a broader understanding of the relationship between chronic pain and PA among older adults with T2D.

## Conclusions

A high prevalence of chronic pain was observed in a register-based sample of older adults with T2D. In addition to frequent and severe chronic pain, factors such as age, BMI, autonomous motivation and feelings of energy were related to being physically active in this patient group. However, among the physically active, pain intensity and frequency did not differentiate between engaging in exercise regularly and engaging only in incidental PA. Instead, factors such as gender, perceived health and autonomous motivation seem to be associated with the likelihood of exercising regularly. Findings support the idea that comprehensive assessments of pain should be used in diabetes care among older adults with T2D.
